# Inherited, not acquired, Gitelman syndrome in a patient with Sjögren’s syndrome: importance of genetic testing to distinguish the two forms

**DOI:** 10.1007/s13730-017-0271-4

**Published:** 2017-08-17

**Authors:** Eikan Mishima, Takayasu Mori, Eisei Sohara, Shinichi Uchida, Takaaki Abe, Sadayoshi Ito

**Affiliations:** 1 Division of Nephrology, Endocrinology, and Vascular Medicine, Tohoku University Graduate School of Medicine, Sendai, Japan; 2 Department of Nephrology, Graduate School of Medical and Dental Sciences, Tokyo Medical and Dental University, Tokyo, Japan; 3 Division of Medical Science, Tohoku University Graduate School of Biomedical Engineering, Sendai, 980-8574 Japan

**Keywords:** SLC12A3, Sodium chloride cotransporter, Exon skip, Hypokalemia, Hereditary kidney disease, Hypomagnesemia

## Abstract

Gitelman syndrome (GS) is an autosomal recessive, salt-losing renal tubulopathy caused by mutations in the *SLC12A3* gene; however, it can also be acquired in patients with autoimmune disease, especially in those with Sjögren’s syndrome. Differentiating between the inherited and acquired forms of GS is clinically difficult. We report a case of inherited, not acquired, GS in a patient with Sjögren’s syndrome. A 41-year-old woman, who had been diagnosed with Sjögren’s syndrome at 27-years-old, had shown chronic hypokalemia (2.5–3.5 mmol/L). Laboratory tests showed hypokalemic alkalosis, hypomagnesemia, and hypocalciuria, corresponding to GS. Although acquired GS associated with Sjögren’s syndrome was initially suspected, a genetic test identified a novel homozygous mutation of c.1336-2A > T in the *SLC12*A3 gene, which resulted in aberrant splicing in the *SLC12*A3 transcript with the exclusion of exons 11 and 12. Thus, the GS was diagnosed as not the acquired but the inherited form. In the diagnosis of GS in patients with autoimmune disease, genetic testing of *SLC12A3* is essential for differentiating the two forms.

**Electronic supplementary material:**

The online version of this article (doi:10.1007/s13730-017-0271-4) contains supplementary material, which is available to authorized users.

## Introduction

Gitelman syndrome (GS) is an autosomal recessive, salt-losing renal tubulopathy characterized by hypokalemic alkalosis, hypomagnesemia, hypocalciuria, and secondary hyperaldosteronism [1]. It is caused by loss-of-function mutations in the *SLC12A3* gene, which encodes thiazide-sensitive sodium chloride cotransporter (NCC) expressed at distal convoluted tubules. Although GS is an inherited disorder, it can on rare occasions be acquired in patients with autoimmune disease [2], especially those with Sjögren’s syndrome.

Sjögren’s syndrome is a systemic autoimmune disease affecting primarily the lacrimal and salivary grands with resultant dryness of the eyes and mouth. In ~10% of patients, renal involvement can occur as an extraglandular manifestation [3]. The most common renal involvement is tubulointerstitial nephritis, which can be revealed by electrolyte disturbances. In addition, in patients with Sjögren’s syndrome, acquired GS has been infrequently reported [4, 5]. Since the first report of such a case by Casatta et al. [2], acquired GS in patients with autoimmune disease has been increasingly described in the literature [4–9].

Although its precise pathogenesis is not clear, immunosuppressive therapy may be potentially useful for acquired GS [2, 5]. Thus, differentiating between acquired and inherited GS is important. However, this is clinically difficult because of their very similar clinical findings, including the same characteristic electrolyte disturbances [10]. Here, we report a case of Sjögren’s syndrome with GS that was diagnosed as not acquired but inherited, which was confirmed by genetic testing and transcript analysis.

## Case report

A 41-year-old woman was admitted to our department for the evaluation of chronic hypokalemia. There was no family history of electrolyte disturbances and no consanguinity. At the age of 21, she had been diagnosed with autoimmune hepatitis and Hashimoto’s disease, and had been treated with oral prednisolone and levothyroxine. At the age of 27, she had been diagnosed with systemic sclerosis by the findings of limbic skin sclerosis, Raynaud’s phenomenon, skin biopsy, and positivity for anti-scl-70 antibody. At the same time, she had been diagnosed with Sjögren’s syndrome with sicca, positivity for antibodies to SSA/Ro, evidence of Schirmer’s test (1 mm/5 min), and ocular surface staining including in the Rose Bengal test.

The chronic hypokalemia had been observed at least from the age of 27, at which the Sjögren’s syndrome had been diagnosed. The serum potassium level had remained between 2.5 and 3.5 mmol/L despite oral supplementation with 32 mmol potassium chloride in the form of four tablets of Slow-K^®^ per day. Laboratory tests (listed in Table 1) showed metabolic alkalosis, hypokalemia with high urinary potassium excretion, hypomagnesemia, low urinary calcium excretion, and secondary hyperaldosteronism with normal blood pressure (100/60 mmHg). Levels of urinary β2-microglobulin and *N*-acetyl-β-d-glucosaminidase, which are markers of tubular damage, were not elevated. She did not report any vomiting, diarrhea, anorexia nervosa, or abuse of diuretics or laxatives. The electrolyte disturbances were highly suggestive of GS. Concerning the underlying diseases, we first suspected that the GS was the acquired form associated with autoimmune diseases.

**Table 1:** Laboratory results of the patient

Laboratory data		(Normal)
Blood		
Urea nitrogen (mg/dL)	11	
Creatinine (mg/dL)	0.5	
Sodium (mmol/L)	137	
Potassium (mmol/L)	3.0	
Chloride (mmol/L)	93	
Calcium (mg/dL)	10.0	
Phosphate (mg/dL)	2.9	
Magnesium (mg/dL)	0.9	
pH	7.451	
Bicarbonate (mmol/L)	32.6	
Renin activity (ng/mL/h)	16.2	(0.3–2.9)
Aldosterone (pg/mL)	167	(30–159)
Urine		
Potassium (mmol/gCr)	72	
Calcium (mg/dL)	<1	
Magnesium (mg/gCr)	77	
Protein (g/gCr)	0.1	
β2 microglobulin (mg/L)	167	(<230)
NAG (IU/L)	7.2	(0.7–11.2)
Serological		
SSA (Ro) Ab (U/mL)	>1200	(<10)
SSB (La) Ab (U/mL)	3.0	(<10)
Scl-70 Ab (U/mL)	>850	(<10)
Centromere Ab (U/mL)	159	(<10)
Antinuclear Ab	1:640	(<1:40)

**Fig. 1:**
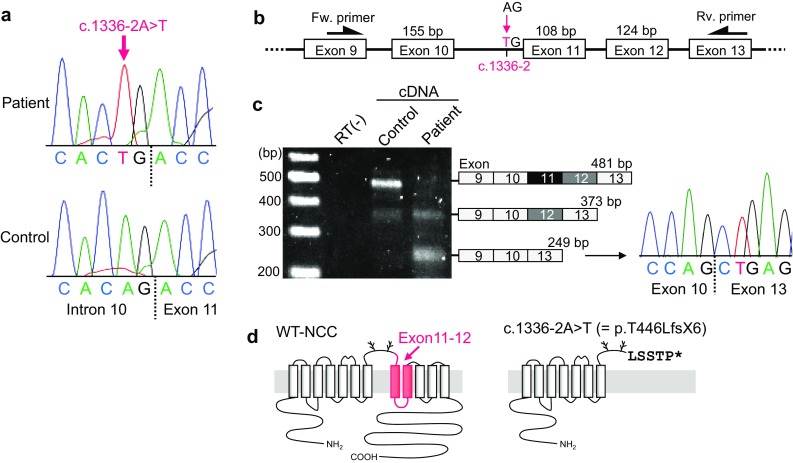
Mutation analysis of c.1336-2A > T in *SLC12A3* gene and mRNA. **a** Genomic DNA sequence in the *SLC12A3* gene of the patient and a healthy control. **b** Locations of the mutation c.1336-2A > T and primers used for the transcript analysis. **c** Reverse-transcription PCR analysis using the cDNA derived from leukocytes. The shown sequence is the aberrant spliced junction of exons 10 and 13. **d** Predicted topology of the wild-type (WT) human sodium chloride cotransporter (NCC) and that of c.1336-2A > T mutated NCC. Sequences deleted as a result of the exon exclusion in the patient are indicated in red. Excluded exons 11 and 12 correspond to the 8th and 9th transmembrane domains

To determine whether the GS was truly acquired or inherited, we performed a genetic testing of *SLC12A3*. Genomic DNA isolated from peripheral blood of the patient was examined by comprehensive genetic testing for major inherited kidney disease genes including *SLC12A3* and *CLCNKB* using a next-generation sequencer (SPEEDI-KID) [11]. Targeted 166 genes are listed in Supplemental Table. The analysis identified a novel homozygous mutation of c.1336-2A > T in *SLC12A3* (NM_000339.2), which was confirmed by Sanger sequencing (Fig. 1a). To exclude the possibility that the c.1336-2A > T is heterozygous mutation with deletion of the other allele at this locus, we additionally performed copy number variation (CNV) analysis using next-generation sequencing data and CONTRA [12]. As a result, we did not find any large deletions or insertions in* SLC12A3* of the patient, confirming the c.1336-2A > T is bi-allelic homozygous mutation.

Because the mutation site is located in the 3′ splice site of intron 10 in *SLC12A3* (Fig. 1b), it is predicted to cause aberrant splicing of the transcript. To confirm the effects of the mutation on the *SLC12A3* transcript, we examined the cDNA derived from the patient’s leukocytes. The region between exon 9 and exon 13 was amplified by PCR, and the amplified fragments were sequenced (Fig. 1c). We found that the mutation resulted in disappearance of the intact transcript and aberrant splicing that excluded exons 11 and 12, resulting in a frameshift and subsequent premature termination (p.T446LfsX6) (Fig. 1d). Thus, the mutation that disrupts the structure of the NCC protein was considered to be a novel causative mutation of GS. To correct the hypokalemia and hypomagnesemia, treatment with spironolactone and magnesium oxide was started, in addition to the potassium supplementation.

## Discussion

We report a case of GS in a patient with Sjögren’s syndrome that was not the acquired but the inherited form. Genetic analysis revealed a novel homozygous mutation, c.1336-2A > T in *SL12A3*, resulting in exon skipping of the transcript. Distinguishing acquired GS from inherited GS is clinically important because the formation of circulating autoantibodies against tubular transporter was suspected to be involved in the pathogenesis [5], raising the possibility that immunosuppressive treatment would be efficacious against the acquired form [2, 5]. Although several cases of acquired GS associated with autoimmune disease have been described [2, 4–9], some of them lacked a genetic test of *SLC12A3* in diagnosing the acquired GS [2, 6, 8]. However, as in the present case, patients with inherited GS can incidentally be complicated with autoimmune diseases because the prevalence of heterozygosity for *SLC12A3* mutation was reported to be approximately 1% in Caucasian population, making GS one of the most common inherited renal tubular disorders [13]. Thus, genetic testing is essential for determining whether GS is acquired or inherited.

Distinguishing the two forms of GS using only the clinical presentation is difficult because the laboratory findings are almost the same, such as hypokalemic alkalosis, hypomagnesemia, and hypocalciuria [10]. Despite GS typically being an inherited disorder, it is usually diagnosed during adolescence or adulthood because of its mild phenotype [13]. The diuretic loading test, which is used for the diagnosis of GS, also does not discriminate between its two forms because acquired GS shows a blunted response to thiazide diuretics, the same as inherited GS [4]. Thus, for the accurate diagnosis of cases with GS and autoimmune disease, genetic testing should be performed.

When genetic testing identifies mutations in the *SLC12A3* gene of GS patients, we should consider whether the mutation is causative. The present mutation, c.1336-2A > T, has not been reported in the literature on patients with GS and has not been listed in either Human Gene Mutation Database [14] or ClinVar [15]. Furthermore, this is an extremely rare variant with no registration in any of the following minor allele frequency databases including Exome Aggregation Consortium [16], 1000 Genome [17], and Integrative Japanese Genome Variation Database [18]. Since the mutation is located in the 3′ splice site for mRNA splicing, we confirmed its influence by transcript analysis. This analysis demonstrated the skipping of exons 11 and 12, disrupting the membrane topology of NCC. In addition, c.1336-1G > C mutation in *SLC12A3*, which is the base adjacent to c.1336-2 located in the same splice site, was previously reported in a patient with GS [19]. Thus, we conclude that the homozygous c.1336-2A > T is a novel causative mutation of GS.

Type III Batter syndrome, which is caused by mutations in the *CLCNKB* gene encoding chloride channel Kb, clinically and biochemically overlaps with GS [13, 19]. Hence, when no mutations are observed in *SLC12A3*, *CLCNKB* gene screening is needed to determine the genetic cause of GS or to rule out inherited GS. In the present case, comprehensive genetic testing using a next-generation sequencer demonstrated no other mutations in over 150 genes (Supplementary Table), including *CLCNKB*, associated with major hereditary kidney diseases in the patient’s genomic DNA.

Our case possessed the extremely rare variant of c.1336-2A > T in *SLC12A3* in homozygous form. There are three possibilities regarding the inheritance pattern of this variant: (1) derivation from heterozygous parents who may have the same ancestor, such as due to the founder effect, (2) de novo mutation, and (3) the patient having the heterozygous variant with deletion of the other allele at this locus. Although sequencing of samples from the parents would resolve the issue of whether the mutation is derived from the parents or occurred de novo, we could not obtain DNA samples from the parents. Thus, both of these possibilities remain open. The CNV analysis ruled out the other possibility of the patient being heterozygous with deletion of the other allele at this locus.

In the present analysis, c.1336-2A > T caused two patterns of aberrant transcripts derived from skipping of only exon 11 and that of exons 11 and 12 (Fig. 1c). Mutations at single splice junctions can result in the skipping of single or multiple exons, creating a cryptic exon by a cryptic splice site, and/or intron retention [20]. Multiexon skipping due to a mutation at a splice junction is occasionally observed in inherited diseases including Gitelman syndrome [21, 22]. In addition, a single mutation can concurrently result in a variety of aberrant transcripts derived from the skipping of a single exon and multiple exons [21]. In the present cDNA analysis, the control sample of a healthy subject showed a transcript with exon 11 spliced out, although the amount was little (Fig. 1c). A recent transcriptomic study has revealed that, in the human body, various patterns of alternative splicing produce numerous alternative transcripts in addition to the full-length or canonical ones [23]. In addition, mis-splicing has also been reported to occur even without mutations, producing a nonfunctional transcript [23]. Thus, in the control sample, the slight presence of spliced out transcripts was conceivable, although they may have no function.

In conclusion, for the accurate diagnosis of GS in patients with autoimmune disease, genetic testing is essential for differentiating the inherited and acquired forms of GS. If only clinical findings are used without genetic testing, an incorrect diagnosis of the cause of GS may be reached.

## Methods

The genetic test was performed in accordance with the ethical standards of Tohoku University Graduate School of Medicine and Tokyo Medical and Dental University. Isolation of genomic DNA and the creation of cDNA were performed as described previously [24]. Primers used for PCR were as follows: exon 9 forward, 5′- ccatttcctacctggccatctc; exon 13 reverse, 5′-gatgagggcataggagcagagg.

## Electronic supplementary material

Below is the link to the electronic supplementary material.

Supplementary material 1 (PPTX 42 kb)
