# Supplementary material for: Inherited, not acquired, Gitelman syndrome in a patient with Sjögren’s syndrome: importance of genetic testing to distinguish the two forms
Source: CEN Case Rep. 2017 Aug 17;6(2):180–4. doi: 10.1007/s13730-017-0271-4 (PMC5694408; doi:10.1007/s13730-017-0271-4)
Supplement: Supplementary file 1 — Supplementary material 1 (PPTX 42 kb) [file 13730_2017_271_MOESM1_ESM.pptx]

## Slide 1
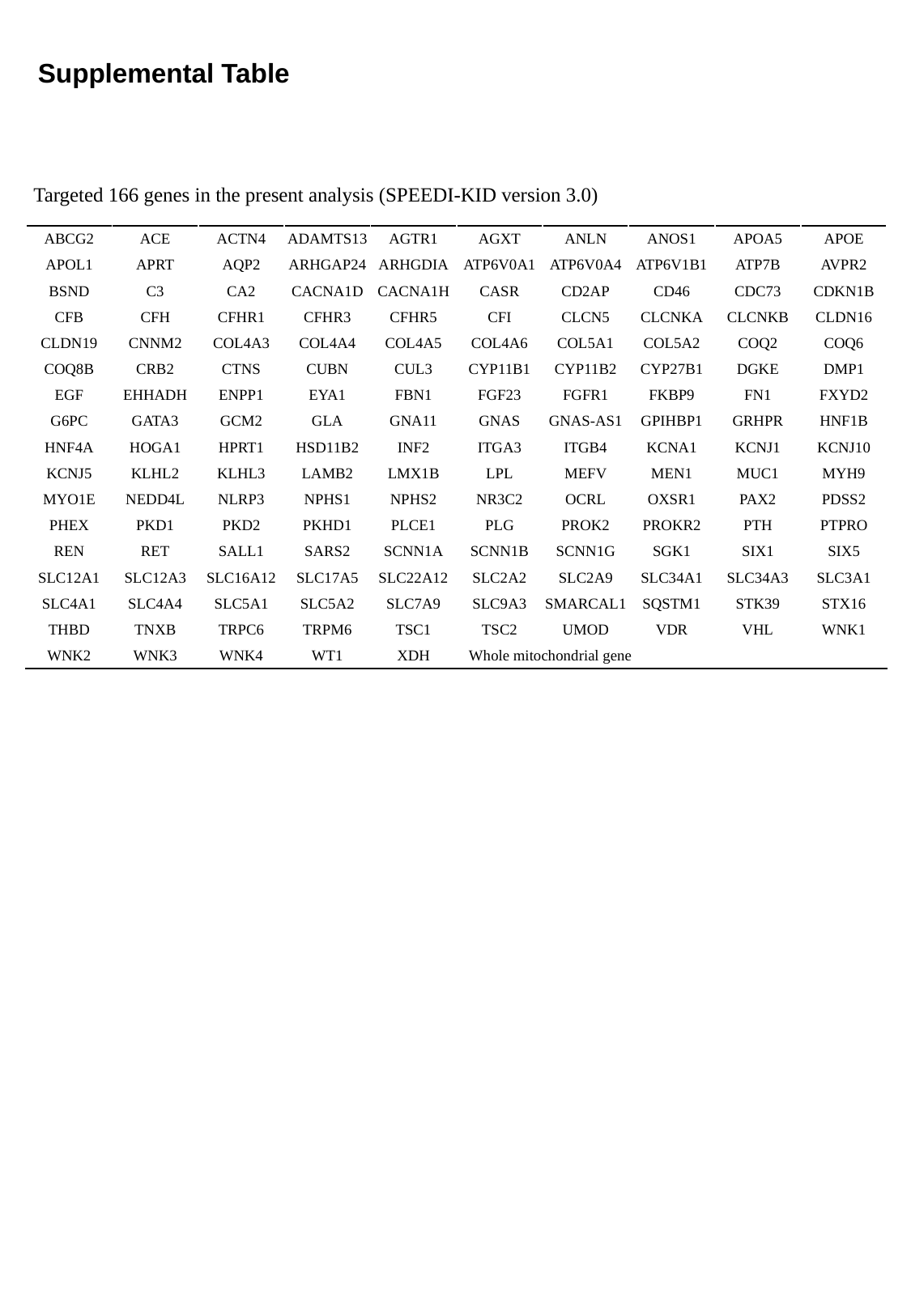

Supplemental Table
Targeted 166 genes in the present analysis (SPEEDI-KID version 3.0)
| ABCG2 | ACE | ACTN4 | ADAMTS13 | AGTR1 | AGXT | ANLN | ANOS1 | APOA5 | APOE |
| --- | --- | --- | --- | --- | --- | --- | --- | --- | --- |
| APOL1 | APRT | AQP2 | ARHGAP24 | ARHGDIA | ATP6V0A1 | ATP6V0A4 | ATP6V1B1 | ATP7B | AVPR2 |
| BSND | C3 | CA2 | CACNA1D | CACNA1H | CASR | CD2AP | CD46 | CDC73 | CDKN1B |
| CFB | CFH | CFHR1 | CFHR3 | CFHR5 | CFI | CLCN5 | CLCNKA | CLCNKB | CLDN16 |
| CLDN19 | CNNM2 | COL4A3 | COL4A4 | COL4A5 | COL4A6 | COL5A1 | COL5A2 | COQ2 | COQ6 |
| COQ8B | CRB2 | CTNS | CUBN | CUL3 | CYP11B1 | CYP11B2 | CYP27B1 | DGKE | DMP1 |
| EGF | EHHADH | ENPP1 | EYA1 | FBN1 | FGF23 | FGFR1 | FKBP9 | FN1 | FXYD2 |
| G6PC | GATA3 | GCM2 | GLA | GNA11 | GNAS | GNAS-AS1 | GPIHBP1 | GRHPR | HNF1B |
| HNF4A | HOGA1 | HPRT1 | HSD11B2 | INF2 | ITGA3 | ITGB4 | KCNA1 | KCNJ1 | KCNJ10 |
| KCNJ5 | KLHL2 | KLHL3 | LAMB2 | LMX1B | LPL | MEFV | MEN1 | MUC1 | MYH9 |
| MYO1E | NEDD4L | NLRP3 | NPHS1 | NPHS2 | NR3C2 | OCRL | OXSR1 | PAX2 | PDSS2 |
| PHEX | PKD1 | PKD2 | PKHD1 | PLCE1 | PLG | PROK2 | PROKR2 | PTH | PTPRO |
| REN | RET | SALL1 | SARS2 | SCNN1A | SCNN1B | SCNN1G | SGK1 | SIX1 | SIX5 |
| SLC12A1 | SLC12A3 | SLC16A12 | SLC17A5 | SLC22A12 | SLC2A2 | SLC2A9 | SLC34A1 | SLC34A3 | SLC3A1 |
| SLC4A1 | SLC4A4 | SLC5A1 | SLC5A2 | SLC7A9 | SLC9A3 | SMARCAL1 | SQSTM1 | STK39 | STX16 |
| THBD | TNXB | TRPC6 | TRPM6 | TSC1 | TSC2 | UMOD | VDR | VHL | WNK1 |
| WNK2 | WNK3 | WNK4 | WT1 | XDH | Whole mitochondrial gene | | | | |
